# MiR-135b-5p is an oncogene in pancreatic cancer to regulate GPRC5A expression by targeting transcription factor KLF4

**DOI:** 10.1038/s41420-022-00814-y

**Published:** 2022-01-13

**Authors:** Daren Liu, Yun Jin, Jinhong Wu, Huanbing Zhu, Dan Ye

**Affiliations:** Department of General Surgery, Second Affiliated Hospital, Zhejiang University, Hangzhou, China

**Keywords:** Cancer, Molecular biology

## Abstract

KLF4 is implicated in tumor progression of pancreatic cancer, but the molecular regulatory mechanism of KLF4 needs to be further specified. We aimed to probe molecular regulatory mechanism of KLF4 in malignant progression of pancreatic cancer. qRT-PCR or western blot was completed to test levels of predicted genes. Dual-luciferase and chromatin immunoprecipitation (ChIP) assays were designed to validate binding between genes. Cell viability and oncogenicity detection were used for in vitro and vivo functional assessment. KLF4 was a downstream target of miR-135b-5p. KLF4 could regulate GPRC5A level. MiR-135b-5p was notably increased in cancer cells, and overexpressing KLF4 functioned a tumor repressive role, which could be restored by miR-135b-5p. Besides, cell malignant phenotypes could be inhibited through reducing miR-135b-5p level, but they were restored by GPRC5A. Our results stressed that KLF4, as a vital target of miR-135b-5p, could influence promoter region of GPRC5A, thus affecting the malignant progression of pancreatic cancer.

## Introduction

Pancreatic cancer, a highly malignant disease of digestive tract, is known as “the king of cancer” due to its latency, difficulty in early diagnosis, low surgical resection rate, low sensitivity to radiotherapy and chemotherapy, high aggressiveness, high risk of relapse, and poor prognosis [1, 2]. With high tendency of cancer metastases in the early stage, the 5-year survival rate of patients is only 6% [3, 4]. Hence, it is essential to find tumor markers that can assist early diagnosis and clarify potential mechanisms of pancreatic cancer metastasis to improve the prognosis of patients and contribute to new treatment avenues for pancreatic cancer.

MiRNAs are generally considered as important regulators of tumors and have biomarker and diagnostic value [5, 6]. A study manifests that the molecular functional mechanism of miRNAs as oncogenes or tumor suppressors in cells may be completed through degradation of targeted mRNAs or transcriptional inhibition [7]. More and more studies disclosed that miRNAs are tightly related to migration and invasion of pancreatic cancer cells and are abnormally expressed in tumor tissue and cell line. For example, miR-939-5p serves as a novel modulator of ARHGAP4 to affect the proliferation and migration of pancreatic cancer [8]. The abnormity in miR-135b-5p expression is implicated with unfavorable clinical features or prognoses in pancreatic cancer patients [9]. Nonetheless, either the mechanism whereby miR-135b-5p regulates pancreatic cancer or target genes of miR-135b-5p are underexplored.

KLF4 is a zinc finger transcription factor, due to its dual activity of transcriptional activation or mechanism, it participates in cell proliferation, differentiation, and apoptosis [10, 11]. Studies confirmed that there are differences in KLF4 expression in human tumor tissues, including prostate cancer [12], liver cancer [13], and breast cancer [14]. Recent research indicates that KLF4 expression in pancreatic cancer tissue and cells is relatively low to normal pancreatic tissue and cells [15]. Besides, in vivo and in vitro upregulating KLF4 are reported to hinder growth and metastasis of pancreatic cancer cells, while knocking down KLF4 exerting the opposite effect [16].

In addition, KLF4 includes deactivation and transcriptional inhibition domains in N-terminal, it exerts an oncogenic or tumor repressive role by functioning with multiple target genes in various tumor tissues [17]. For instance, in colon cancer, KLF4 inversely modulates IFITM3, and the dysregulation of KLF4 expression results in IFITM3 abnormal expression, which is the cause of progression and metastasis of cancer cells [18]. Our research unveiled that miR-135b-5p was markedly dysregulated in pancreatic cancer, and it could regulate transcription factor KLF4, but the specific molecular mechanism has not been clarified. Therefore, we tried to excavate the genes which are regulated by KLF4 in pancreatic cancer, finding that the promoter region of GPRC5A could be regulated by KLF4. Above all, this research attempted to clarify molecular mechanism whereby miR-135b-5p regulates KLF4/GPRC5A to affect malignant progression of pancreatic cancer cells. Our findings lay a theoretical foundation for new diagnoses and treatments for pancreatic cancer.

## Results

### MiR-135b-5p is upregulated in pancreatic cancer

**Fig. 1:**
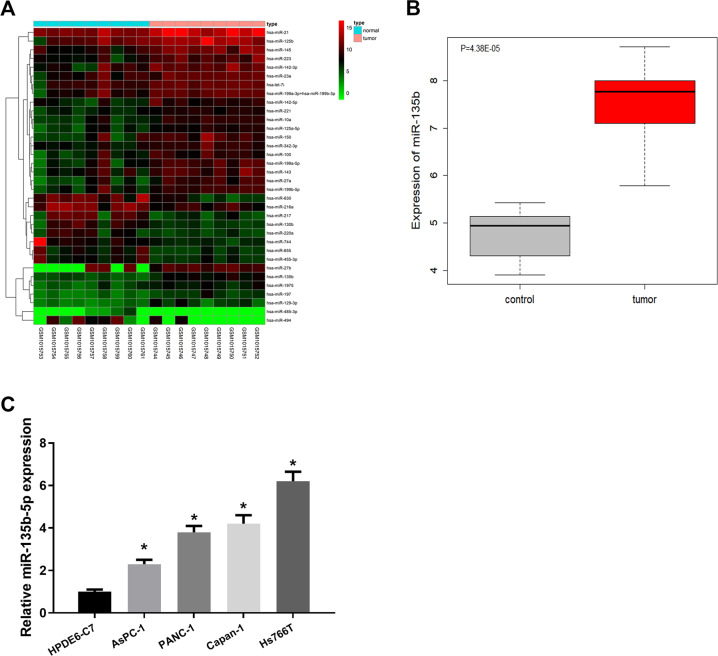
MiR-135b-5p is upregulated in pancreatic cancer cells. **A** Heat map of differentially expressed miRNAs in pancreatic cancer microarray GSE41369, with the horizontal coordinate representing numbers of samples and the vertical coordinate representing names of genes. **B** Box plot of differential miR-135b level in microarray GSE41369, in which the gray box represents normal and red represents tumor samples. **C** Levels of miR-135b-5p in HPDE6-C7, PANC-1, Capan-1, AsPC-1, and Hs766T cells. (* represents *p* < 0.05).

In differential analysis of miRNA expression microarray GSE41369 of pancreatic cancer, 34 miRNAs that were significantly differentially expressed were finally obtained (Fig. 1A). By comparison, we found that among the 34 miRNAs, miR-135b showed significantly high expression in pancreatic cancer with the smallest *p* value of significant difference (Fig. 1B), which indicated that miR-135b was the most notably differentially expressed in pancreatic cancer. Therefore, we believe that miR-135b is likely to play a more vital modulatory role in pancreatic cancer compared with the other 33 miRNAs. MiR-135b-5p is a maturity of miR-135b. Numerous studies unveiled dysregulated miR-135b-5p in multiplex cancers [19, 20]. Accordingly, miR-135b-5p was selected as the research object.

In addition, miR-135b-5p level in pancreatic cancer cell lines was assessed. Compared with HPDE6-C7 cells, miR-135b-5p was markedly increased in PANC-1, Capan-1, AsPC-1, and Hs766T cells (Fig. 1C). To probe modulatory mechanism of miR-135b-5p in pancreatic cancer, we selected two cell lines AsPC-1 (with lower miR-135b-5p) and Hs766T (with higher miR-135b-5p).

### Overexpressing miR-135b-5p promotes malignant progression of pancreatic cancer cells

**Fig. 2:**
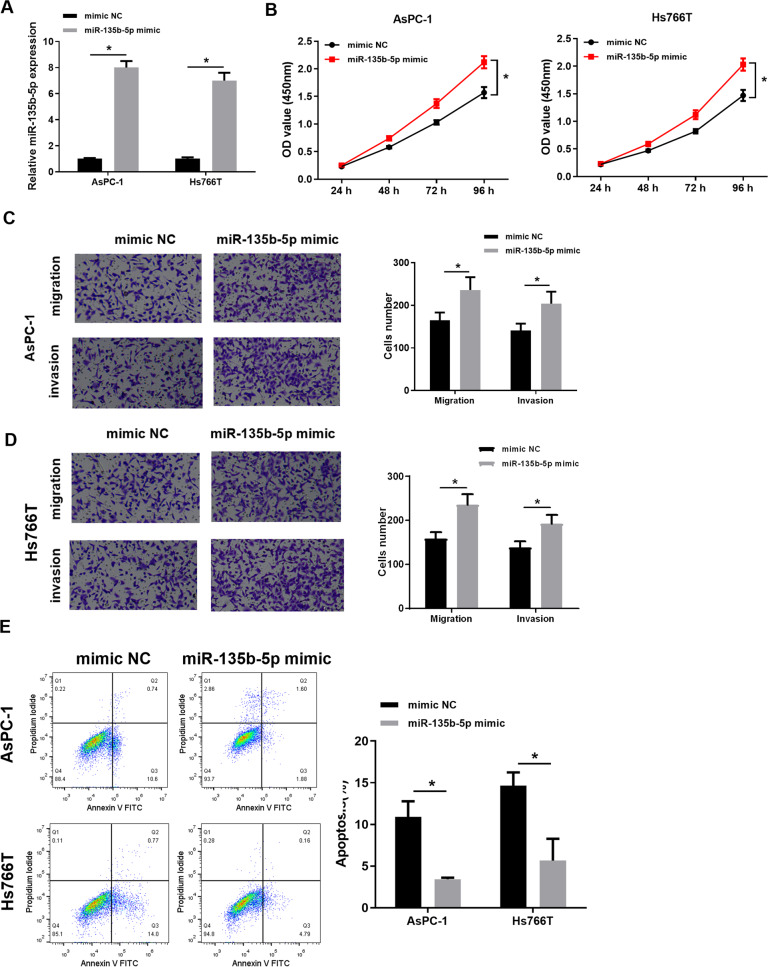
Overexpressing miR-135b-5p fosters malignant progression of pancreatic cancer cells. **A** qRT-PCR was used to assay transfection efficiency of miR-135b-5p in pancreatic cancer cell lines (AsPC-1 and Hs766T). **B** CCK8 assay assayed cell activity of miR-135b-5p mimic and its NC groups. **C** The migratory and invasive potentials of cell line AsPC-1 in each transfection group via Transwell assay (×100). **D** The migratory and invasive potentials of cell line Hs766T in each transfection group through Transwell assay (×100). **E** Flow cytometry was conducted to test apoptosis rate of miR-135b-5p mimic and its NC groups. (* represents *p* < 0.05).

MiR-135b-5p in AsPC-1 and Hs766T were overexpressed. The results manifested that miR-135b-5p in two pancreatic cancer cell lines increased considerably with miR-135b-5p mimic transfection (Fig. 2A). Then, we performed a series of cell function experiments to detect the transfected cells. CCK8 experimental results exhibited that cell proliferative activity was stronger after overexpressing miR-135b-5p (Fig. 2B). The results of Transwell assays displayed that compared with NC, migratory and invasive potentials were also considerably enhanced after overexpressing miR-135b-5p (Fig. 2C, D). In addition, we also conducted apoptosis experiments with flow cytometry and found that overexpressing miR-135b-5p decreased the apoptosis rate of the cells (Fig. 2E). Hence, miR-135b-5p fostered malignant phenotypes of pancreatic cancer cells.

### MiR-135b-5p targets to down-regulate expression of KLF4

**Fig. 3:**
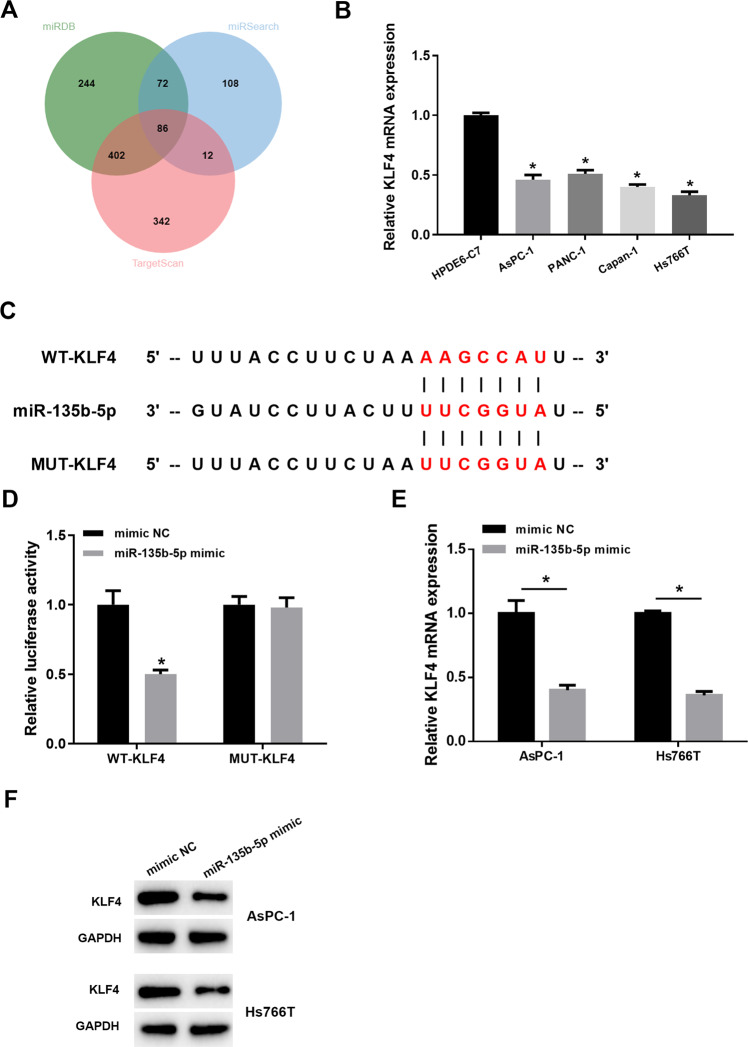
KLF4 is a downstream regulatory target of miR-135b-5p. **A** The downstream target genes of miR-135b were predicted. The three circles in the figure indicate the prediction outcomes of miRDB, miRSearch and TargetScan databases, respectively, and the middle part indicates intersection of three groups of data. **B** KLF4 mRNA level in HPDE6-C7, PANC-1, Capan-1, AsPC-1, and Hs766T cells. **C**, **D** The targeted relationship of miR-135b-5p and KLF4 verified via dual-luciferase assay. **E**, **F** The change of KLF4 level after overexpressing miR-135b-5p assayed through qRT-PCR and western blot assay. (* indicates *p* < 0.05).

To better understand molecular modulatory mechanism of miR-135b-5p, possible targets of miR-135b were predicted through miRDB database, miRSearch database and TargetScan database, and the prediction results of the three databases were intersected (Fig. 3A). Next, 86 genes that may be regulated by miR-135b were finally obtained. Gene interaction analysis was further performed on the 86 targets, and the core value was calculated. It was found that the core value of KLF4 was the highest. Previous studies unraveled that transcription factor KLF4 level in colon cancer and pancreatic cancer is considerably reduced [21–23], but its upstream regulatory factor is still unclear. Therefore, we believe that KLF4 may take a regulatory part in pancreatic cancer as the downstream target of miR-135b-5p.

Then, KLF4 level in pancreatic cancer cells was assayed through qRT-PCR, and it was disclosed that relative to normal cells, KLF4 mRNA level was markedly decreased in cancer cell lines (Fig. 3B). As illustrated in Fig. 3C, D, overexpressing miR-135b-5p weakened luciferase activity of cells with WT-KLF4, while had no significant impact on cells with MUT-KLF4. In addition, KLF4 level in AsPC-1 and Hs766T cells with miR-135b-5p overexpression was also detected via qRT-PCR and western blot assay. KLF4 mRNA and protein levels decreased by upregulating miR-135b-5p (Fig. 3E, F). Therefore, miR-135b-5p decreased KLF4 in pancreatic cancer cells.

### MiR-135b-5p is a controller of malignant behaviors of pancreatic cancer cells via suppressing KLF4

**Fig. 4:**
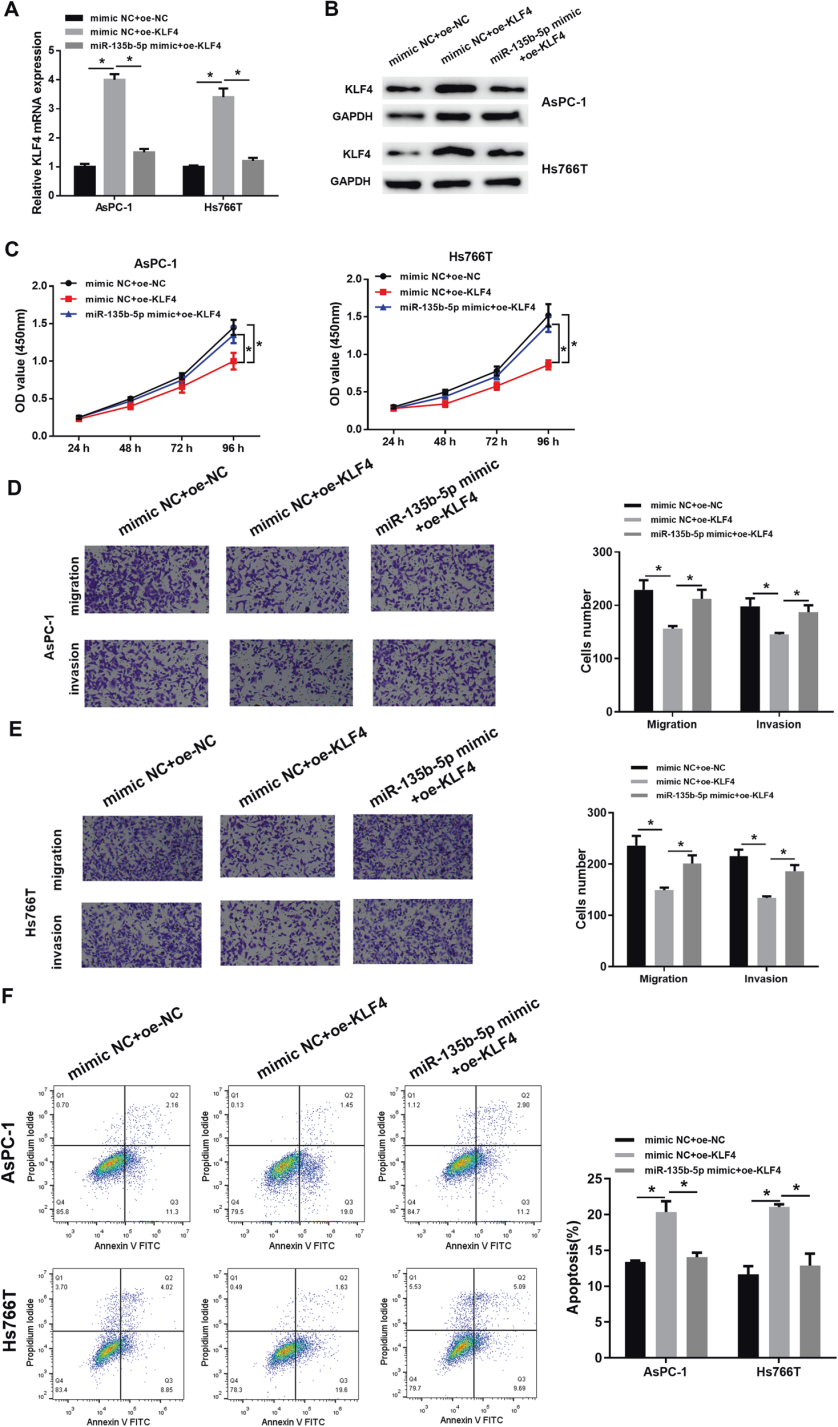
MiR-135b-5p can regulate cell proliferation, migration and invasion of pancreatic cancer by suppressing KLF4. **A**, **B** KLF4 mRNA and protein levels in AsPC-1 and Hs766T cells of each transfection group via qRT-PCR and western blot assay. **C** Cell viability in each group tested through CCK8 assay. **D** The migratory and invasive potentials of the cell line AsPC-1 in each transfection group assayed via Transwell assay (×100). **E** The migratory and invasive potentials of cell line Hs766T in each transfection group assayed through Transwell assay (×100). **F** Flow cytometry tested cell apoptosis rate in each transfection group. (* represents *p* < 0.05).

Meanwhile, to assay whether miR-135b-5p could affect pancreatic cancer cell function by regulating KLF4, we set up 3 cell groups and conducted a series of cell function experiments for verification. KLF4 level in each transfection group was detected through qRT-PCR and western blot assay, and the results manifested that in the transfection groups of AsPC-1 and Hs766T cells, KLF4 overexpression could be restored by miR-135b-5p (Fig. 4A, B). In proliferation experiments, overexpressing KLF4 could inhibit cell proliferative potential. But after continuously overexpressing miR-135b-5p, the proliferative property of cells was considerably enhanced (Fig. 4C). Meanwhile, in the migration and invasion experiments, overexpressing KLF4 also hindered cell migration and invasion, but overexpressing miR-135b-5p could reverse this inhibitory effect (Fig. 4D, E). In addition, it was also observed by flow cytometry that cell apoptosis rate was significantly raised with overexpressing KLF4. However, in the recovery group, cell apoptosis rate was markedly decreased with overexpressing miR-135b-5p (Fig. 4F). In conclusion, miR-135b-5p could hamper KLF4, thus promoting proliferation, migration, and invasion, as well as hindering apoptosis of pancreatic cancer cells.

### Transcription factor KLF4 can negatively modulate GPRC5A level

**Fig. 5:**
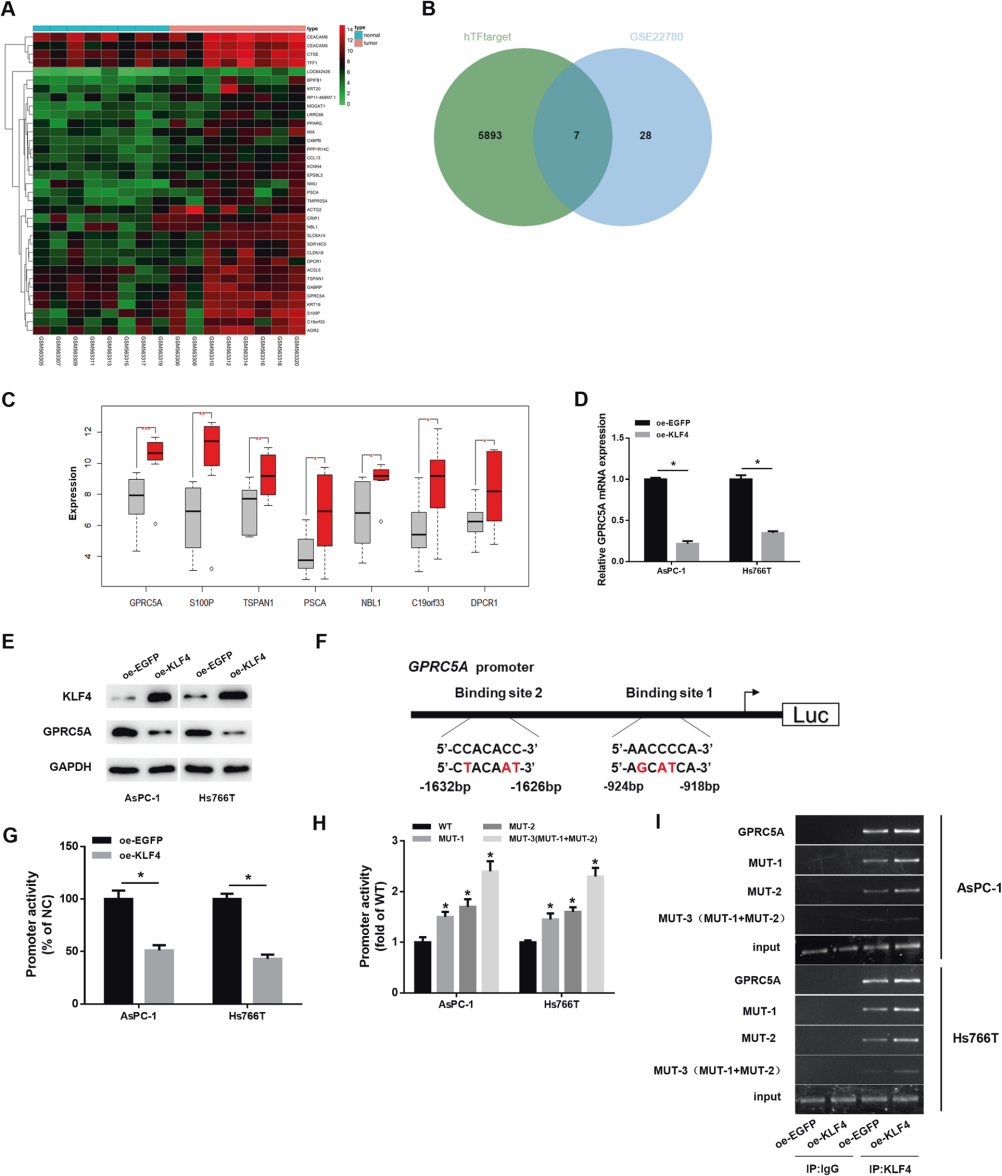
Transcription factor KLF4 can negatively regulate the expression of GPRC5A. **A** Heat map of significantly upregulated genes in microarray GSE22780, with the horizontal coordinate representing the sample numbers and the vertical coordinate representing the gene names. **B** The intersection of the downstream regulatory genes of transcription factor KLF4 on hTFtarget database and the upregulated genes in microarray GSE22780. **C** Box plot of the differential expression of the 7 potential downstream regulatory genes of KLF4 in microarray GSE22780. The gray box indicates normal while the red indicates the tumor samples. **D**, **E** The mRNA and protein expression of GPRC5A in AsPC-1 and Hs766T assayed via qRT-PCR and western blot assay when KLF4 was overexpressed. **F** Schematic diagram of GPRC5A promoter indicates that a GPRC5A promoter and MUTs of GPRC5A are generated in two KLF4 binding sites in AsPC-1 and Hs766T cells. **G**, **H** GPRC5A promoter activity assayed via dual-luciferase assay (MUT-1: mutations at Binding site 1; MUT-2: mutations at Binding site 2; MUT-3: mutations at both binding sites). **I** Specific anti-KLF4 antibodies and IgG antibodies located on the two sides of GPRC5A promoter region were used to perform ChIP experiment, and the promoter region contains the assumed KLF4 binding sites (MUT-1: mutations at Binding site 1; MUT-2: mutations at Binding site 2; MUT-3: mutations at both binding sites). (* indicates *p* < 0.05, ** indicates *p* < 0.01, *** indicates *p* < 0.004).

Many studies have confirmed that KLF4 is a transcription factor related to tumor molecular regulatory mechanism, and we had also confirmed that its differential expression can affect malignant behaviors of cancer cells. We explored the key gene, which was regulated by KLF4 and differentially expressed to understand the regulatory mechanism of KLF4 in pancreatic cancer. We obtained a gene expression microarray GSE22780 of pancreatic cancer from GEO with the aid of bioinformatics analysis, and the differential analysis showed that 35 genes were notably highly expressed in pancreatic cancer (Fig. 5A). Then, downstream regulatory genes of KLF4 were predicted by hTFtarget database. They were intersected with the significantly upregulated genes in microarray GSE22780 (Fig. 5B), and seven genes were found in the intersection. The differential expression of the seven genes in GSE22780 was further analyzed (Fig. 5C), and it was found that GPRC5A was the most differentially expressed in the microarray. Therefore, we believe that GPRC5A may be affected by KLF4 in pancreatic cancer and thus take an essential regulatory part.

To clarify molecular mechanism of KLF4 negatively regulating GPRC5A expression, GPRC5A expression in KLF4 overexpressed group and EGFP overexpressed group of the two pancreatic cancer cell lines was assayed. As demonstrated in Fig. 5D, E, GPRC5A mRNA expression and protein expression both decreased notably after overexpressing KLF4. In addition, site-directed mutagenesis was used to identify the function of KLF4 in transcriptional regulation of GPRC5A, and a GPRC5A promoter and GPRC5A mutations in two KLF4-binding sites in AsPC-1 and Hs766T cells were generated (Fig. 5F). The results indicated that overexpressed KLF4 markedly inhibited the activity of GPRC5A promoter (Fig. 5G), while mutation of KLF4 binding sites on GPRC5A promoter significantly increased the activity (Fig. 5H), which presented that KLF4 binding sites were inverse modulatory elements of GPRC5A promoter. ChIP experiment was performed to assay the way that KLF4 directly interacts with GPRC5A promoter. Results in Fig. 5I indicated that the DNA fragment with the predicted size from immunoprecipitation was amplified in the transfection group with KLF4 antibody. But binding property of mutant GPRC5A promoter with KLF4 noticeably weakened. These results suggested that transcription factor KLF4 could bind to GPRC5A promoter in cancer cells, and overexpressing KLF4 enhanced binding to GPRC5A promoter.

### MiR-135b-5p affects malignant progression of pancreatic cancer cells through KLF4/GPRC5A

**Fig. 6:**
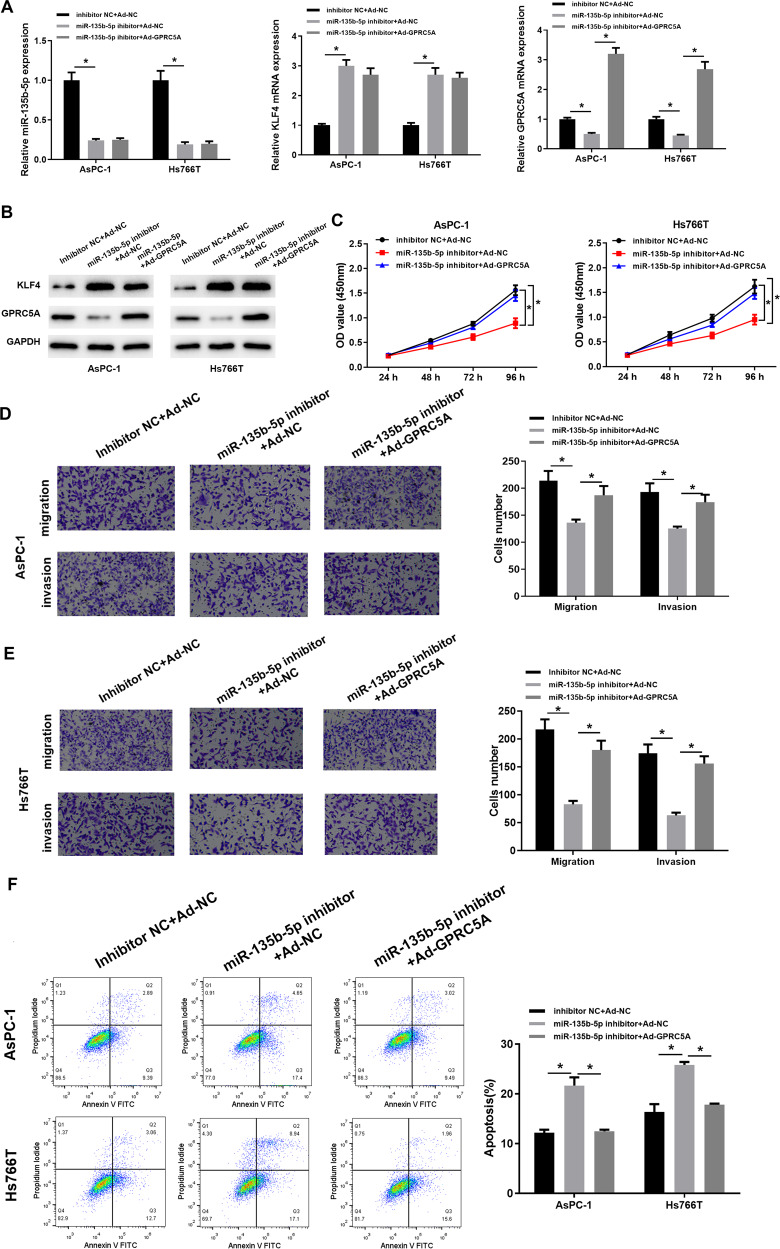
MiR-135b-5p can affect malignant behaviors of pancreatic cancer cells through KLF4/GPRC5A. **A** MiR-135b-5p, KLF4 mRNA and GPRC5A mRNA levels in AsPC-1 and Hs766T cells assayed via qRT-PCR. **B** KLF4 and GPRC5A protein expression in AsPC-1 and Hs766T cells assayed through western blot assay. **C** Cell viability of each transfection group tested via CCK8 assay. **D** The migratory and invasive abilities of the cell line AsPC-1 in each transfection group detected by Transwell assay (×100). **E** The migratory and invasive potentials of the cell line Hs766T in each transfection group assayed via Transwell assay (×100). **F** Cell apoptosis rate in each transfection group tested via flow cytometry. (* indicates *p* < 0.05).

Based on the above results, the effects of miR-135b-5p on GPRC5A level and malignant progression of pancreatic cancer cells via targeting KLF4 were further verified. First, 3 groups of transfected cells were obtained: inhibitor NC+ Ad-NC, miR-135b-5p inhibitor+Ad-NC and miR-135b-5p inhibitor+Ad-GPRC5A, and miR-135b-5p, KLF4 mRNA, and GPRC5A mRNA levels in each cell line were assessed via qRT-PCR. When miR-135b-5p level was repressed, KLF4 level was notably upregulated, and GPRC5A was significantly downregulated. When miR-135b-5p was inhibited and GPRC5A was overexpressed, GPRC5A level was markedly increased, while the other two genes showed no significant changes (Fig. 6A). Western blot assayed KLF4 and GPRC5A protein levels, and the results were also consistent with the above findings (Fig. 6B). This disclosed that miR-135b-5p could modulate GPRC5A in pancreatic cancer cells by targeting KLF4. The proliferation experimental results unveiled that cell proliferative potential was also inhibited after expression of miR-135b-5p was reduced, but the proliferative ability was significantly restored after overexpressing GPRC5A (Fig. 6C). The results of Transwell assay also confirmed that low miR-135b-5p levels inhibited cell migration and invasion, but they were markedly improved after the expression of GPRC5A was increased (Fig. 6D, E). Meanwhile, in the apoptosis experiment, the results were similar to the above. Cell apoptosis rate was increased with a low miR-135b-5p level, but it could be reduced after overexpressing GPRC5A (Fig. 6F). In conclusion, our experimental data demonstrated that low miR-135b-5p level could affect GPRC5A by KLF4, thus hindering cell malignant phenotypes of pancreatic cancer, but the inhibitory effect could be reversed by overexpressing GPRC5A.

### Nude mice experiments verify the impact of miR-135b-5p/KLF4/GPRC5A regulatory axis on tumor growth

**Fig. 7:**
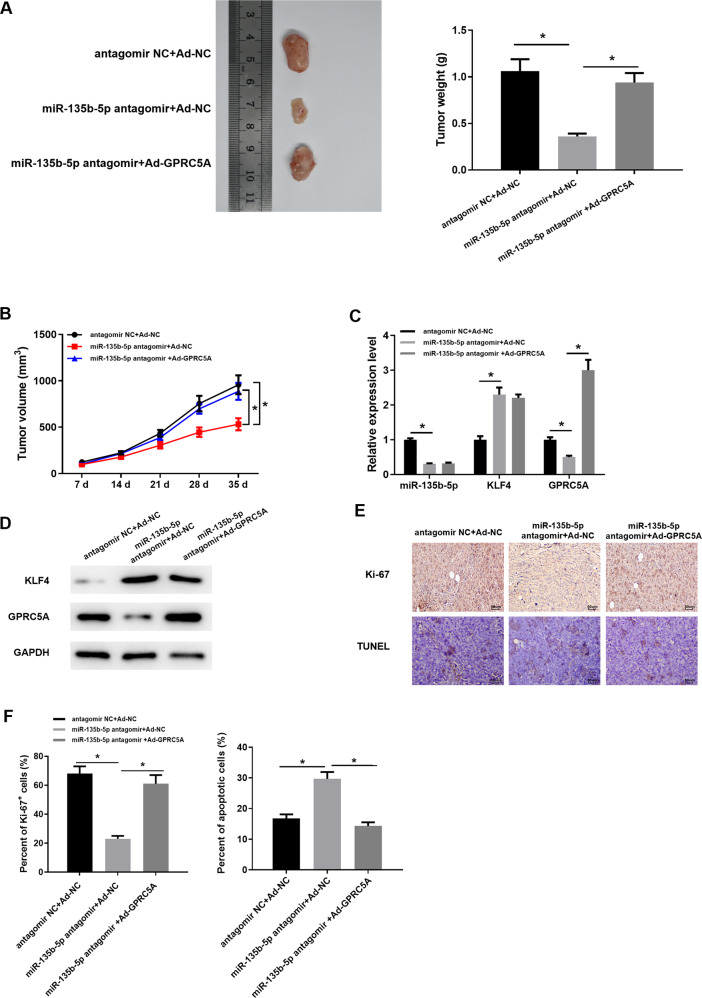
In vivo experiments demonstrate the impact of miR-135b-5p/KLF4/GPRC5A regulatory axis on tumor growth. **A** Tumor formation in nude mice from different treatment groups after 35 d; **B** Tumor sizes were continuously measured in different treatment groups; **C** MiR-135b-5p, KLF4 mRNA and GPRC5A mRNA levels in tumor tissue assayed via qRT-PCR; **D** KLF4 and GPRC5A protein expression in tumor tissue tested by Western blot assay; **E** Expression of Ki-67 in tumor tissue assayed via immunohistochemistry, and cell apoptosis assayed via TUNEL (200×); **F** Statistics of the percentage of Ki-67 positive cells and apoptotic cells. (* represents *p* < 0.05).

Finally, the above findings prompted us to validate regulatory effect of miR-135b-5p on GPRC5A and their effect on tumor growth by in vivo experiments. Three groups of mice were injected with AsPC-1 cell lines of different treatments, respectively. In comparison to NC group, tumor growth in miR-135b-5p antagomir+Ad-NC group was markedly slower, but the speed of tumor growth was significantly restored after overexpressing GPRC5A. It was consistent with the findings in the above cell function experiment (Fig. 7A, B). Then, the tumor tissue was assessed via qRT-PCR. MiR-135b-5p and GPRC5A were conspicuously decreased in the tumor tissue of miR-135b-5p antagomir+Ad-NC group, and the expression of KLF4 was higher than NC group, while GPRC5A level in the miR-135b-5p antagomir+Ad-GPRC5A group was markedly higher than that in the other 2 groups (Fig. 7C). In addition, western blot assay also manifested that KLF4 protein expression was increased in the miR-135b-5p antagomir+Ad-NC group compared with that of the other two groups, while GPRC5A protein expression was notably decreased; besides, GPRC5A protein expression was conspicuously upregulated in miR-135b-5p antagomir+Ad-GPRC5A group (Fig. 7D). These results suggested that tumor growth was mainly dependent on GPRC5A level in cells. Immunohistochemistry and TUNEL staining results also demonstrated that ratio of Ki-67 positive cells decreased markedly and ratio of cell apoptosis increased when GPRC5A was lowly expressed caused by inhibiting miR-135b-5p. Later, when GPRC5A was overexpressed, the results were reversed (Fig. 7E, F). In summary, we validated the impact of miR-135b-5p/KLF4/GPRC5A regulatory axis on pancreatic cancer cells. The low miR-135b-5p level could slow down cell growth of pancreatic cancer in vivo, which could be reversed by overexpressing GPRC5A.

## Discussion

A lot of research teams probed the implication between miR-135b and cancers. MiR-135b is reported as a tumor promoter in breast cancer, colorectal cancer and other cancers [19, 24]. A study reveals that miR-135b is a new biomarker for pancreatic ductal adenocarcinoma [25]. In addition, Zhang Z et al. [26] proposed that miR-135b-5p fosters migration, invasion, and epithelial-mesenchymal transition (EMT) of pancreatic cancer cells via down-regulating the expression of NR3C2. But in the opposite, several studies reported that miR-135b-5p exerts a repressive effect in prostate cancer and glioblastoma [20, 27]. Being consistent with most studies, we validated that miR-135b-5p was upregulated in pancreatic cancer through bioinformatics analysis and cellular experiments, and the results of the cell function experiments suggested that overexpressing miR-135b-5p could stimulate malignant behaviors of pancreatic cancer cells. Collectively, miR-135b-5p exerts an oncogenic role in pancreatic cancer.

KLF4 participates in the occurrence and progression of breast cancer [28], lung cancer [29], prostate cancer [30], colorectal cancer [31] and other cancers. For example, in recent studies, circulating tumor cells (CTC)-iChip technology is applied to purify CTC from pancreatic cancer patients for RNA-seq characterization, displaying the enrichment of main relevant genes in the stem genes LIN28B/KLF4 [32]. KLF4 as a tumor repressor negatively modulates CD44 and restrains metastasis of pancreatic cancer [33]. But a study offers contradictory findings that KLF4 is upregulated in invasive pancreatic cancer cells and human tumor tissues, and facilitates tumor growth in mice [34]. Notwithstanding these inconsistent findings, they display the pivotal role of KLF4 in cancers. In this study, miRDB database, miRSearch database, TargetScan database, and STRING database were used to confirm that KLF4 was the gene with the highest core value in downstream targets of miR-135b, and targeted relationship between miR-135b-5p and KLF4 was validated by dual-luciferase assay. Meanwhile, KLF4 level was decreased in cancer cell lines, and overexpressing miR-135b-5p could inhibit the expression of KLF4. Earlier clinical evidence existed that KLF4 is lowly expressed in pancreatic cancer tissues, particularly in the advanced stage, which is congruous with our results [15]. In addition, cell experiments also suggested that overexpressing KLF4 hindered malignant behaviors of cancer cells, but the inhibitory effect was reduced by miR-135b-5p. In summary, we fully elucidated the negative modulatory relationship of miR-135b-5p and KLF4, as well as regulatory mechanism linking this relationship to pancreatic cancer cells. Interestingly, miR-135b-5p level was noticeably different between AsPC-1 and Hs766T cells, but there was no significant difference in the expression of KLF4. The reasons may be that KLF4 is not only regulated by miR-135b-5p in pancreatic cancer cells, but also be regulated by other genes and cytokines. For example, phosphorylated STAT3 can upregulate KLF4 level to maintain stemness of pancreatic cancer cells [35]. But this supposition and the specific reasons need confirmation in follow-up research.

Many studies have demonstrated that KLF4 modulates various genes at the transcriptional level. For example, Hu W et al. [36] found that KLF4 serves as a tumor repressive gene in lung cancer by hindering hTERT and MAPK signaling transmission. To inquiry about the molecular mechanism of KLF4 expression in pancreatic cancer progression, we explored the downstream genes of KLF4. HTFtarget database was employed for prediction of downstream regulatory genes of KLF4, and notably upregulated 35 genes in microarray GSE22780 were then intersected with the predicted genes, and the most obvious difference in pancreatic cancer was found in the expression of GPRC5A. A study found abnormal GPRC5A in a variety of malignant diseases, suggesting the involvement of GPRC5A in the progression of tumors [37]. Sawada Y et al. [38] found that GPRC5A affects bone metastasis of prostate cancer via facilitating cell proliferation. As such, our results indicated that GPRC5A was upregulated in pancreatic cancer, and upregulated expression of KLF4 also inhibited the expression of GPRC5A. More importantly, dual-luciferase assay manifested that KLF4 could directly bind GPRC5A promoter elements as well as negatively regulate transcriptional expression of GPRC5A. Both in vivo and in vitro experiments verified that miR-135b-5p could affect malignant progression of pancreatic cancer through KLF4/GPRC5A.

In conclusion, this study provided significant insights into the miR-135b-5p/KLF4/GPRC5A axis in pancreatic cancer progression. MiR-135b-5p fostered malignant behaviors of pancreatic cancer cells by decreasing KLF4, while KLF4 hampered the disease by negatively modulating the transcriptional expression of GPRC5A. This study unveiled molecular mechanism of miR-135b-5p/KLF4/GPRC5A axis in human pancreatic cancer cells and demonstrated the probability of miR-135b-5p as a new therapeutic target. Furthermore, we plan to collect clinical tissue samples to clarify the implication between the regulatory axis and clinicopathological features or prognosis of pancreatic cancer patients, thus providing novel markers for early diagnosis or prognosis of pancreatic cancer patients.

## Methods

### Bioinformatics methods

The expression microarrays GSE41369 and GSE22780 of pancreatic cancer were obtained from Gene Expression Omnibus (GEO) database (https://www.ncbi.nlm.nih.gov/geo/). GSE41369, a miRNA expression microarray, includes 9 normal samples and 9 tumor samples. GSE22780, a gene expression microarray, includes 8 controls and 8 tumor samples. The R-language “limma” package was used for differential analysis. The screening criteria of the differentially expressed genes was |logFC| > 2, *p* < 0.05. Then, miRNAs and genes with significantly differential expression in pancreatic cancer were gained. The downstream target genes of miR-135b were predicted through miRDB, miRSearch, and TargetScan databases. Through STRING database, gene interaction analysis on candidate targets of miR-135b was conducted, and core value of each gene was counted. The core value refers to the ability of a gene to interact with other genes. The more the interacting genes, the higher the core value of this gene. Through consulting on hTFtarget database, the downstream regulatory genes of KLF4 were predicted.

### Cell lines and culture conditions

Human pancreatic duct epithelial cell line HPDE6-C7 (BNCC338285) and pancreatic cancer cell lines PANC-1 (BNCC277096), Capan-1 (BNCC337700), AsPC-1 (BNCC277096), and Hs766T (BNCC350780) were all provided with BeNa Culture Collection (Beijing, China). All of them were cultured in DMEM (Gibco, USA) plus 10% fetal bovine serum (FBS; Gibco, USA), supplemented with an appropriate amount of streptomycin (100 mg/mL; Gibco, USA) and penicillin (100 units/mL; Gibco, USA) in a constant temperature incubator at 37 °C and 5% CO_2_.

### Cell transfection

MiR-135b-5p inhibitor, miR-135b-5p antagomir, miR-135b-5p mimic, ORF Lentiviral expression clone for KLF4 (oe-KLF4) and GPRC5A (Ad-GPRC5A), and their control mimic NC, inhibitor NC, antagomir NC, oe-NC, oe-EGFP (enhanced green fluorescent protein) and Ad-NC were accessed from GeneCopoeia (Rockville, MD, USA). Lipofectamine 2000 (Thermo Fisher Scientific, Inc.) was implemented to transiently transfect pancreatic cancer cells per instructions. Cells were prepared in corresponding medium under routine conditions for future use. Before transfection, all cells should be cultured in a complete medium for at least 24 h and rinsed with phosphate buffer saline (PBS, pH 7.4) before transient transfection.

### Total RNA extraction and qRT-PCR

Total RNA isolation from tissue or cells was completed by TRIzol (Life Technologies, USA) reagent. NanoDrop 2000 system (Thermo Fisher Scientific, Inc., USA) was employed to determine the RNA concentration. According to kit instructions of miScript II RT Kit (Qiagen, USA) and PrimeScript RT Master Mix (Takara, P.R. China), miRNA and mRNA were transcribed into cDNA, respectively. MiR-135b-5p level was measured with miScript SYBR Green PCR Kit (Qiagen, Germany), while KLF4 mRNA and GPRC5A mRNA levels were determined with SYBR ^®^ Premix Ex Taq TM II (Takara Bio Inc., Japan). Applied Biosystems® 7500 Real‐Time PCR Systems (Thermo Fisher Scientific, MA) were recommended for qRT-PCR and assessment of miR-135b-5p, KLF4, and GPRC5A levels. MiR-135b-5p took U6 as internal reference. KLF4 and GPRC5A took GAPDH as an endogenous reference. The 2^−ΔΔCt^ method was employed for quantification. The primers are listed in Table 1.

**Table 1:** Primer sequence.

Gene	Sequence (5′–3′)
miR-135b-5p	F: GGTATGGCTTTTCATTCCT
	R: CAGTGCGTGTCGTGGAGT
U6	F: GGAGCGAGATCCCTCCAAAAT
	R: GGCTGTTGTCATACTTCTCATGG
KLF4	F: CCCACATGAAGCGACTTCCC
	R: CAGGTCCAGGAGATCGTTGAA
GPRC5A	F: ATGGCTA CAACAGTCCCTGAT
	R: CCACCGTTTCTAGGACGATGC
GAPDH	F: GGAGCGAGATCCCTCCAAAAT
	R: GGCTGTTGTCATACTTCTCATGG

### Western blot assay

Cells were rinsed 3 times with cold PBS and added with radioimmunoprecipitation assay (RIPA) lysis buffer (RIPA; Pierce, Rockford, IL, USA) on ice for 10 min. A BCA kit (Thermo Fisher Scientific, Waltham, MA, USA) was implemented for protein concentration determination. Polyacrylamide gel electrophoresis was conducted for 20 μg of the total proteins, which were then electrophoresed onto PVDF (Amersham, USA) membrane. Then the blocking solution was poured. Membrane was incubated with primary antibodies against KLF4 (abcam, UK), GPRC5A (abcam, UK) and GAPDH (abcam, UK) overnight at 4 °C. Then, the membrane was rinsed by PBST (PBS with 0.1% Tween-20) 3 times (10 min/time). Then, secondary antibody goat anti-rabbit IgG H&L (abcam, UK) labeled with horseradish peroxidase was added. The membrane was rinsed with PBST buffer for 3 times, and 10 min each time. Optical luminometer (GE, USA) was used for scanning and development, followed by capture of images.

### CCK-8 assay

After 48 h of cell transfection, trypsin digestion and cell resuspension were conducted. The 96-well plates were recommended for cell inoculation (5 × 10^3^ cells/well) and cell culture condition was an incubator at 37 °C and 5% CO_2_. At a specific time point (24, 48, 72, 96 h), 10 μL CCK-8 (Dojindo Laboratories, Mashiki-machi, Kumamoto, Japan) of 5 mg/mL concentration was supplemented, followed by 2 h of cell incubation. Absorbance at 450 nm was assayed by FLx800 Microplate Reader (BioTek, Winooski, VT, USA).

### Transwell migration and invasion assays

Non-matrix coated/ Matrigel (BD Biosciences, Franklin Lakes, NJ, CA, USA) coated Transwell inserts were recommended to assess migratory and invasive potentials of cells. The upper chamber was supplemented with cells (2 × 10^4^ cells/mL) plus 200 μL serum-free DMEM, and the lower chamber was filled with DMEM with 10% FBS (700 μL). Twenty-four h later, cells on the surface of upper insert were gently removed with PBS and a cotton swab. After migratory and invasive cells were subjected to 4% paraformaldehyde for 20 min of fixing at room temperature, they were subjected to 0.1% crystal violet for 10 min of staining at room temperature. Five fields were chosen randomly for counting and photographing by a fluorescence microscope (Olympus Corporation, Tokyo, Japan).

### Cell apoptosis assay

To assay the apoptosis of AsPC-1 and Hs766T cells, double staining by Annexin V-fluorescein isothiocyanate (FITC) /propidium iodide (PI) was used in this study. After 48 h of culture of transfected cells (2 × 10^4^ cells/mL), they were rinsed twice with PBS, centrifuged and suspended in binding buffer. Annexin V-FITC/PI was recommended to process cell suspension from light for 15 min. Finally, a flow cytometer (Becton and Dickinson Company, Franklin Lakes, New Jersey, USA) was implemented for determination of apoptotic cells, and the data were subjected to CELLQuest 3.0 software (Becton and Dickinson Company) for analysis.

### Dual-luciferase reporter gene assay

Dual-luciferase assay was conducted to validate whether KLF4 level could be modulated via miR-135b-5p. The synthesized KLF4 mutant (MUT) and wild-type (WT) 3’UTR were connected to the downstream of pmiRGLO luciferase vectors (Promega, WI, USA) to generate WT-KLF4 and MUT-KLF4, which were co-transfected into HEK-293T cells with miR-135b-5p mimics or NC mimics, respectively. After cell culture and incubation in DMEM plus 10% FBS for 48 h, DUAL-Glo assay kit (Promega, Madison, WI, USA) was recommended for assay of firefly and renilla luciferase expression.

### GPRC5A promoter activity analysis

GPRC5A promoter with a length of about 500 bp was cloned to pGL3 alkaline luciferase reporter vector. The GPRC5A promoter was mutated by QuikChange site-directed mutagenesis kit (Stratagene). DNA sequencing was done to identify the mutation. Activity of GPRC5A promoter was determined through luciferase assay. After 24 h of transfection, luciferase activity was assayed.

### Chromatin immunoprecipitation assay (CHIP)

AsPC-1 and Hs766T cells were inoculated in a culture dish (15 cm) and oe-KLF4 or oe-EGFP was transfected to these cells. The GPRC5A promoter was mutated by QuikChange site-directed mutagenesis kit (Stratagene). ChIP test was performed with a ChIP kit (Cell Signaling Technology). Following DNA-protein crosslinking, cells were lysed in sodium dodecyl sulfate (SDS) lysis buffer. The DNA of the lysate was cut by ultrasonic to about 500 bp. KLF4 antibody (abcam, UK) was added for co-immunoprecipitation overnight at 4 °C. Normal rabbit IgG antibody (abcam, UK) was used as negative control. Protein A-Sepharose was used to extract DNA within 2 h. Then, the promoter region was amplified by PCR to about 500 bp, and the PCR product was electrophoresed on 2% agarose gel.

### Experiments on animals

Eighteen female BALB/c nude mice (4-5 weeks) were accessed from National Laboratory Animal Center (Beijing, China). Mice were stochastic assign into 3 groups: antagomir NC+Ad-NC, miR-135b-5p antagomir+Ad-NC, and miR-135b-5p antagomir+Ad-GPRC5A groups, with 6 mice in each group. The stably transfected pancreatic cancer cells (1 × 10^6^) were subcutaneously injected on the right side to detect their tumorigenicity ability. Tumor growth was observed and recorded by taking photos. The size of the tumor was recorded every 7 days, and the volume of the tumor was plotted as a growth curve. Five weeks later, nude mice were killed, and tumor weight and size were measured. Tumor tissue was taken for subsequent experiments.

### Immunohistochemical method and TUNEL staining

Anti-Ki67 antibody (abcam, UK) was used in immunohistochemical analysis for the tumor tissue overnight at 4 °C. Normal rabbit IgG secondary antibody (abcam, UK) was supplemented and tissue was placed at 37 °C for 60 min. Then, diaminobenzidine (DAB) was recommended for color development reaction. Counterstaining of nucleus was done by using hematoxylin. In addition, TUNEL staining was completed with In Situ Cell Death Detection Kit (Roche Group, Basel, Switzerland). Staining was observed under an optical microscope (Olympus) at magnification of ×200.

### Data analysis

All the data were processed by GraphPad Prism (La Jolla, CA) version 6.0 statistical software. Measurement data were presented in the form of mean ± standard deviation. *T* test was conducted for comparison between two groups. *P* < 0.05, *p* < 0.01 and *p* < 0.004 mean that the difference reaches statistically significant.

## Data Availability

All data generated or analyzed during this study are included.
